# Cohort Profile: The Northern Sweden Health and Disease Study (NSHDS)

**DOI:** 10.1093/ije/dyaf004

**Published:** 2025-02-03

**Authors:** Florentin Späth, Patrik Wennberg, Robert Johansson, Lars Weinehall, Margareta Norberg, Anna Rosén, Gerd Johansson, Anna Nordström, Ingegerd Johansson, Lena Maria Nilsson, Sture Eriksson, Anna Winkvist, Maria Wennberg, Sophia Harlid, Sara Rebbling, Beatrice Melin, Olov Rolandsson, Malin Sund, Ingvar A Bergdahl, Stefan Söderberg, Göran Hallmans, Bethany Van Guelpen

**Affiliations:** Department of Diagnostics and Intervention, Oncology, Umeå University, Umeå, Sweden; Department of Public Health and Clinical Medicine, Umeå University, Umeå, Sweden; Department of Public Health and Clinical Medicine, Umeå University, Umeå, Sweden; Department of Epidemiology and Global Health, Umeå University, Umeå, Sweden; Department of Epidemiology and Global Health, Umeå University, Umeå, Sweden; Department of Diagnostics and Intervention, Oncology, Umeå University, Umeå, Sweden; Department of Public Health and Clinical Medicine, Umeå University, Umeå, Sweden; Department of Medical Sciences, Uppsala University, Uppsala, Sweden; Department of Sport Sciences, UiT The Arctic University of Norway, Tromsø, Norway; Department of Odontology, Umeå University, Umeå, Sweden; Department of Epidemiology and Global Health, Umeå University, Umeå, Sweden; Department of Public Health and Clinical Medicine, Umeå University, Umeå, Sweden; Department of Community Medicine and Rehabilitation, Geriatric Medicine, Umeå University, Umeå, Sweden; Department of Public Health and Clinical Medicine, Umeå University, Umeå, Sweden; Department of Public Health and Clinical Medicine, Umeå University, Umeå, Sweden; Department of Diagnostics and Intervention, Oncology, Umeå University, Umeå, Sweden; Biobanken Norr, Region Västerbotten, Umeå, Sweden; Department of Diagnostics and Intervention, Oncology, Umeå University, Umeå, Sweden; Department of Public Health and Clinical Medicine, Umeå University, Umeå, Sweden; Department of Diagnostics and Intervention, Surgery, Umeå University, Umeå, Sweden; Department of Surgery, University of Helsinki and Helsinki University Hospital, Helsinki, Finland; Department of Public Health and Clinical Medicine, Umeå University, Umeå, Sweden; Department of Public Health and Clinical Medicine, Umeå University, Umeå, Sweden; Department of Public Health and Clinical Medicine, Umeå University, Umeå, Sweden; Department of Diagnostics and Intervention, Oncology, Umeå University, Umeå, Sweden; Wallenberg Centre for Molecular Medicine, Umeå University, Umeå, Sweden

**Keywords:** prospective cohort, population-based study, longitudinal cohort, prospective blood samples, biobank, NSHDS, disease risk, risk factor, lifestyle intervention, biomarkers

Key Features

- The Northern Sweden Health and Disease Study (NSHDS) was initiated in the mid-1980s. The NSHDS is a population-based prospective longitudinal cohort comprising >140 000 participants in the two northernmost regions in Sweden, Norrbotten and Västerbotten, with >240 000 blood samples and 1.5 million person-years of follow-up.
- The NSHDS includes three sub-cohorts: the Västerbotten Intervention Programme (VIP), the expanded Northern Sweden Monitoring of Trends and Determinants of Cardiovascular Disease (MONICA) Study, and the Mammography Screening Project (MSP). The VIP is both a community-based cardiometabolic intervention programme encouraging healthy lifestyle (targeting individuals 40, 50, and 60 years of age), and a corresponding research cohort. The MONICA is an observational study focusing on cardiovascular disease and its associated risk factors, recruiting individuals aged 25–74 years. The MSP recruited women attending mammography during 1995–2006. The NSHDS median participation age is 50 years (53% women).
- Most participants contribute data on health, lifestyle, anthropometric measures, blood pressure, blood lipids, and glucose tolerance, along with research blood samples that are fractionated, frozen within an hour of collection, and stored at –80°C. Linkage to registries, clinical cohorts, and biological tissue archives facilitates studies of well-characterized participants (often combined with intervention studies).
- Collaborations are encouraged. Additional information can be found at: info.brs@umu.se; https://www.umu.se/en/biobank

## Why was the cohort set up?

During the 1980s, cardiovascular disease rates were disproportionately high in northern Sweden [1]. To improve the cardiometabolic health of the population and to encourage research on aetiology, prevention and early detection of disease, the Västerbotten Intervention Programme (VIP), was initiated in 1985. The VIP is a community intervention focusing on cardiovascular disease and diabetes prevention through population low-risk and individual high-risk strategies. It includes longitudinal risk factor screening, as well as lifestyle counselling and other individual prevention measures. Primary healthcare centres invite residents from specific age groups for health examinations and risk factor measurements, initially in selected municipalities, region-wide since 1991. The intervention protocol is described in detail elsewhere [2]. The corresponding VIP research cohort includes frozen research blood samples, in addition to data from the questionnaires and measurements collected at the health examinations.

Concurrent with the initiation of the VIP cohort in 1985, the two northernmost regions in Sweden, Norrbotten and Västerbotten, joined the multinational WHO Monitoring of Trends and Determinants of Cardiovascular Disease (MONICA) Study, aiming to follow time trends in cardiovascular morbidity, mortality, and its risk factors [3–5]. To further strengthen the research resources generated by the VIP and the base MONICA cohort, the MONICA cohort was expanded beyond the original international protocol to include, for example, a separate collection of blood samples and expanded questionnaires on lifestyle habits. Additionally, to increase both the number of women and longitudinal blood samples, the Mammography Screening Project (MSP) was conducted during 1995–2006, including women who attended mammography in Västerbotten.

The VIP research cohort, the expanded MONICA, and the MSP together comprise the Northern Sweden Health and Disease Study (NSHDS) [4, 6]. The NSHDS was initiated with a cancer focus, to complement the cardiovascular disease focus of the VIP and MONICA cohorts. However, the design of the NSHDS facilitates research on a broad range of life-science topics and disease endpoints. The design, data and blood sample collection, handling and storage protocols of the NSHDS subcohorts have been managed together for nearly four decades.

The NSHDS and its subcohorts contribute to several major international collaborations and consortia. One such long-standing research collaboration is the European Prospective Investigation into Cancer and Nutrition (EPIC) including >500 000 participants across 10 European countries [7]. Another example is the participation of Northern Sweden MONICA in the Monica Risk Genetics Archiving and Monograph (MORGAM) study [8].

The NSHDS research blood samples are stored in a centralized biobank (Biobanken Norr), within the general healthcare system (Region Västerbotten). The Section of Biobank and Registry Support within the Department of Public Health and Clinical Medicine (Umeå University) is responsible for managing the NSHDS data. The NSHDS is supported by the Umeå University Faculty of Medicine, Region Västerbotten and Region Norrbotten (the public healthcare providers in these two regions), external grants, and fees for data and sample extraction.

## Who is in the cohort?

The NSHDS is a population-based, prospective, longitudinal cohort that continues to recruit participants through the VIP and MONICA subcohorts. As of May 2020, 141 910 individuals were enrolled in the NSHDS, with a median age of 50 years and 53% women (Table 1). The period up to May 2020 was selected as it represents the most appropriate timeframe for describing the population-based cohort. Since then, recruitment and data collection in the VIP have been substantially lower due to disruptions from the COVID-19 pandemic and increased strain on primary healthcare.

**Table 1.** Selected characteristics of the NSHDS participants by subcohort

Characteristic	Full cohort	Subcohorts		
	NSHDS	VIP	MONICA	MSP
Participants, *n* (%)				
All	141 910^a^	127 069	12 298	28 822
Women	75 042 (52.9)^a^	64 227 (50.5)	6242 (50.8)	28 822 (100.0)
Men	66 868 (47.1)^a^	62 842 (49.5)	6056 (49.2)	0 (0)
Observations, *n*	272 682	202 070	16 210	54 402
Median age,^b^ years (IQR)	50.2 (19.8)	50.0 (19.7)	51.2 (22.0)	58.2 (10.8)
Median blood pressure,^b^ mmHg (IQR)				
Systolic	125 (21)	125 (21)	127 (28)	‒
Diastolic	81 (16)	81 (16)	80 (15)	‒
Median blood lipids,^b^ mmol/l (IQR)				
Total cholesterol	5.4 (1.5)	5.4 (1.5)	5.8 (1.6)	‒
HDL cholesterol	1.4 (0.6)	1.4 (0.6)	1.4 (0.5)	‒
LDL cholesterol	3.4 (1.4)	3.4 (1.4)	3.2 (1.5)	‒
Triglycerides	1.1 (0.8)	1.1 (0.8)	1.3 (0.8)	‒
Oral glucose tolerance test,^b^ %				
Elevated fasting plasma glucosec	15.4	15.4	12.5	‒
Elevated 2-h plasma glucosec	7.3	7.1	14.2	‒
Body mass index,^b^ %				
Underweight (<18.5)	0.7	0.7	0.7	0.9
Healthy weight (18.8‒24.9)	42.7	42.4	40.4	44.8
Overweight (25.0‒29.9)	39.6	39.9	41	38
Obesity (≥30.0)	16.9	16.1	17.9	16.3
Smoking,^d^ %				
Never smoker	51.9	52.1	49.3	‒
Previous smoker	31.4	31.3	30.2	‒
Current smoker	16.7	16.6	20.5	‒
Alcohol use (AUDIT score),^d^ %				
Low risk (0–7)	‒	93.7	‒	‒
Medium risk (8–15)	‒	5.8	‒	‒
High risk (16–19)	‒	0.3	‒	‒
Addiction likely (20–40)	‒	0.2	‒	‒
University degree,^d^ %	30.5	31.1	23	‒

AUDIT, Alcohol Use Disorders Identification Test; HDL, high-density lipoprotein; LDL, low-density lipoprotein; MONICA, Monitoring of Trends and Determinants of Cardiovascular Disease Study; MSP, Mammography Screening Project; VIP, Västerbotten Intervention Programme.

a Indicating the number of unique study participants (excluding overlap between subcohorts).

b Age, blood pressure, blood lipids, blood glucose, and BMI values calculated for each participant using all available data.

c Elevated fasting glucose: ≥6.1 mmol/l (VIP and MONICA); elevated 2-h glucose: ≥8.9 mmol/l in VIP (capillary plasma) and ≥7.8 mmol/l in MONICA (venous plasma).

d Values for smoking, alcohol use, and education represent data from the latest available observation.

**Figure 1.**
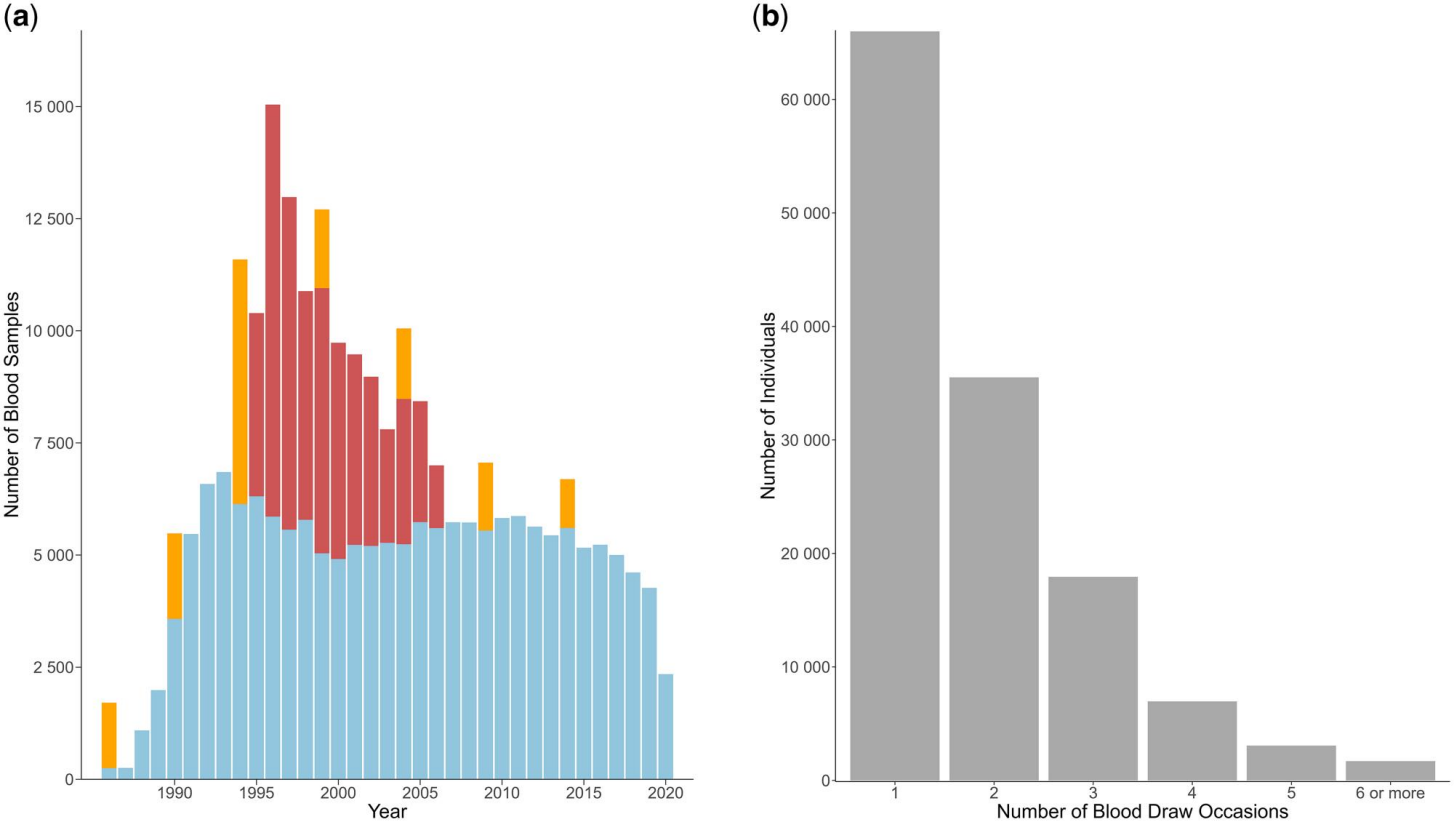
The number of blood samples in the NSHDS in (a) by calendar year and sub-cohort: the Northern Sweden MONICA study (orange), the MSP (red), and the VIP (blue) and in (b) the number of blood draw occasions per participant in the NSHDS.

The recruitment of participants for the NSHDS differs by subcohort. In the VIP, study enrolment and the collection of data and blood samples are integrated into general healthcare. Every year, residents of Västerbotten who turn 30 (until 1995), 40, 50, and 60 years old are invited to a health examination and cardiometabolic risk factor screening at their local primary healthcare centre. The health examination is followed by individualized counselling based on motivational interviewing methodology with a trained nurse to promote healthy lifestyle and reduce the risk of developing hypertension, diabetes, and cardiovascular disease. Follow-up visits and/or referrals to the local general practitioner are provided according to guidelines. The Northern Sweden MONICA employs cross-sectional recruitment. Since 1986, the MONICA invites residents of Norrbotten and Västerbotten to participate in the survey, with subsequent surveys conducted approximately every five years [4, 5, 9]. On each occasion, 2500 individuals aged 25–74 years are randomly invited to participate (2000 individuals aged 26–64 in 1986 and 1990). Data and blood sample collection, handling, and storage in the MONICA adhere to protocols very similar to the VIP. The MSP recruited women attending mammography in Västerbotten during 1995–2006. Blood samples and limited questionnaire data were collected at the mammography centre after the procedure. Overall, 18.5% (*n *=* *26 279) of individuals in the NSHDS participated in more than one subcohort. Approximately 45% (*n *=* *64 000) of all participants in the NSHDS underwent repeated sampling occasions (Fig. 1), and 70% (*n *=* *45 000) of these occurred within the VIP.

**Figure 2.**
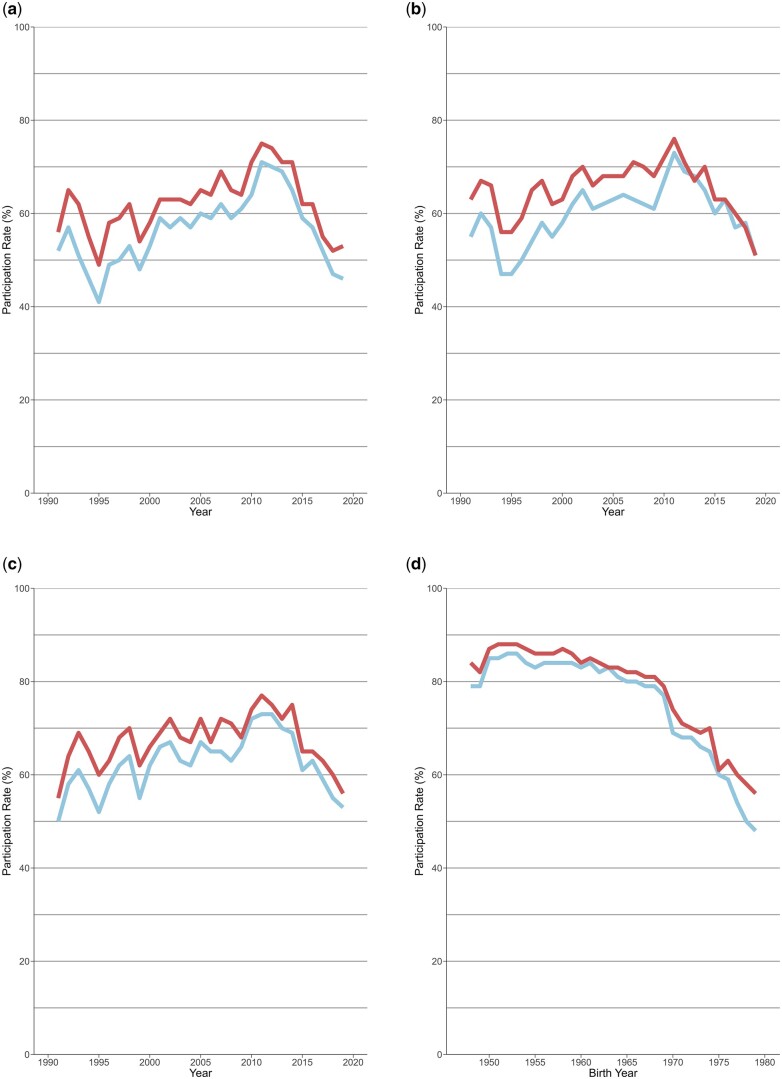
Participation rates in the VIP in women (red) and men (blue) in (a) invited at 40 years of age, in (b) at 50 years of age, in (c) at 60 years of age, and in (d) by birth year and regardless of whether individuals participated one or more times (i.e. the curves in (d) represent individuals who participated on at least one occasion and participation rates are therefore highest among earlier birth cohorts). Participation rates were calculated by dividing the number of the VIP participants by the number of the eligible population (i.e. all residents in Västerbotten of the same sex and age in each recruitment year).

Invitations to the VIP are intended for the entire population but vary over time and between healthcare centres due to staffing and/or financial constraints. Data on the number of individuals specifically invited to the VIP and MSP subcohorts are not available. Therefore, we calculated participation rates in relation to the eligible population (i.e. all residents in Västerbotten of same sex and age in each year). For the VIP, this yielded annual participation rates of approximately 50%–80% (Fig. 2). The participation rate in the MSP was lower and estimated to be approximately 29% of all women living in Västerbotten in the eligible population for mammography screening during recruitment. In the MONICA, the median participation rate across surveys was 74% among those invited. Participation in the VIP and the MONICA is lower in younger individuals and has declined across all age groups since approximately 2012 in the VIP and 2009 in the MONICA (Fig. 2 and Supplementary Fig. S1). This report includes data until May 2020, thus the impact of the COVID 19 pandemic on the data presented is negligible.

The representativeness of the NSHDS was evaluated in earlier studies, revealing minor differences [10–12]. Nonparticipation in the VIP correlated weakly with unemployment/lower income, single marital status, younger age, and being born outside of Sweden [10, 11]. Despite these disparities, it is important to note that the VIP, the largest of the NSHDS subcohorts, is population-based [13]. Similar to the VIP, the MONICA nonparticipants were younger and more likely to smoke, have diabetes, be single, and less likely to have a university degree [12].

## How often have they been followed up?

Since the expansion of the VIP to cover the entire region in 1991, approximately 5000–7000 health examinations and risk factor measurements have been performed annually. In May 2020, the VIP database comprised 127 069 unique individuals. Of these, 31% (*n *=* *39 740) participated twice, 13% (*n *=* *16 810) participated three times at 10-year intervals, and 55% (*n *=* *70 230) participated once. The MONICA is primarily cross-sectional by design, but in 1999, all 6276 previously invited, living individuals were re-invited (66% participation). During 2016–2019, 802 participants in the MONICA who had participated in two previous MONICA surveys and attained an age of 80 years were invited to the Silver-MONICA follow-up study, to gather exposure data for gerontological research (68% participation). In the MSP, follow-up was typically possible every two years (breast cancer screening), and 51% (*n *=* *28 800) participants have more than one sampling occasion. Altogether, 141 910 participants in the NSHDS, as of May 2020, collectively contributed >240 000 blood samples for research (Fig. 1).

Loss to follow-up due to emigration or withdrawal of consent are rare events and have been observed in 0.6% and in <0.2% of all participants in the NSHDS, respectively. Thus, for the acquisition of outcome data, essentially complete follow-up is possible. Despite the large amount of data requested from each participant in the NSHDS, the proportion of missing data is low and often related to structural factors such as changes in the questionnaires over time. The proportion of participants in the VIP and the MONICA with available questionnaire data, health examination measurements, and a blood sample is 84%.

## What has been measured?

### Measurements

Measurements within the NSHDS differ by subcohort and have been described previously [5, 6]. Briefly, in the VIP and the MONICA, height, weight, waist circumference, and blood pressure are measured by health care staff according to standardized procedures. Blood lipid levels and fasting glucose levels are analyzed in the VIP and the MONICA participants. In addition, a standard oral glucose tolerance test is administered in individuals without known diabetes (replaced by haemoglobin A1c in the VIP since 2021). Additional details, protocol changes, and calibration history are available online (https://www.umu.se/en/biobank). In the MSP, the participants’ height and weight were collected [6]. The measurement data available in the NSHDS are summarized in Table 2.

**Table 2.** Examples of incident cases with prospective blood samples in the NSHDS

Characteristic	**Single** ^a^ ***n***	**Repeated** ^a^ ***n***	**Total** ***n***
Blood samples			
Plasma (EDTA and heparin)	65 100	64 300	129 400
Erythrocytes	63 600	63 100	126 700
Buffy coat/DNA	64 300	63 600	127 900
Measures			
Anthropometric measures^b^	67 400	71 200	138 600
Blood glucose levels	71 100	40 700	111 800
Blood lipid levels	73 500	44 100	117 600
Blood pressure	73 400	44 300	117 700
Questionnaire data^c^	67 500	71 600	139 100
Cardiovascular disease^d^^,^^e^			
Ischemic heart disease	3610	2540	6150
Stroke	3810	3200	7010
Diabetes^d^^,^^e^			
Diabetes type I	80	30	110
Diabetes type II	1240	710	1950
Cancer^d^^,^^f^			
Prostate cancer	2570	1760	4330
Breast cancer	1330	1950	3280
Colorectal cancer	1340	1360	2700
Urothelial and kidney cancer	870	610	1480
Lung cancer	650	620	1270
Pancreatic cancer	270	300	570
Haematological malignancies	1050	950	2000

a Data for prospective single and repeated measures are shown.

b Including height, weight, and waist circumference.

c Including, e.g. sociodemographic data, education, medical history, family history, health and lifestyle data, dietary, and quality of life assessment.

d Indicates the number of individuals with either prospective single or repeated blood samples in the NSHDS.

e All diagnoses are verified and detailed clinical data, e.g. medical history, medication, and disease phenotype data are extracted from patient records by trained staff.

f Data available from the cancer register include, e.g. cancer diagnosis date, tumour-specific data regarding tumour site, histological type, stage, and follow-up data.

### Questionnaire data and dietary assessment

Participants in the VIP and the MONICA are invited to complete a comprehensive questionnaire, including socioeconomic and psychosocial factors, social support, working conditions, physical activity, alcohol intake, tobacco consumption, quality of life, and personal and family health history, particularly focusing on cardiometabolic health [5]. In the MSP, women were invited to complete a brief questionnaire focusing on reproductive factors.

Dietary data are collected in the VIP and the MONICA by a validated semi-quantitative food frequency questionnaire on common food items and dishes reflective of the habitual dietary intake during the preceding year [14]. Portion sizes are indicated on four colour pictures, and intake frequency ranges from never to more than four times per day. Daily intakes of energy and nutrients are calculated by linkage to the national food composition database of the Swedish Food Agency. All dietary data collected and refined in the NSHDS comprise the Northern Sweden Diet Database (NSDD).

Data collection in the VIP and the MONICA are currently modernized, including the implementation of digital questionnaires.

### Blood samples collected for future research

In all the NSHDS subcohorts (the VIP, the MONICA, and the MSP), >90% of participants provided blood samples for future research. Samples are collected in the morning in the VIP, generally after overnight fasting, and throughout the day in both the MONICA and the MSP. The fasting time was >8 h for 67% of all the NSHDS samples and 91% of the VIP samples. The samples are collected by venepuncture in 10-ml tubes—one with EDTA, one with heparin, and, in the MONICA, also one with serum. Each sample is aliquoted into five fractions: three plasma, one buffy coat, and one vial with erythrocytes. The needle-to-freezer time is <1 h. Samples are initially frozen at –20°C, and within 1 week of collection, all samples are in long-term storage in –80°C freezers. The sample processing protocol has remained the same throughout the study period.

### Sources of morbidity data

Measures, variables, and blood samples available within the NSHDS are summarized in Table 2. To exemplify some types of accessible endpoint data, we linked the NSHDS to several registries: the MONICA registries (MONICASE) [4], providing record-validated diagnoses of myocardial infarction and stroke; the Diabetes Register in Northern Sweden (DiabNorth) [15], providing record-validated diabetes diagnoses in the VIP and the MONICA; and the National Cancer Register [16]. Participants with both single and repeated pre-diagnostic blood samples and who were later diagnosed with cardiovascular disease, diabetes, and cancer are shown in Table 2. Through the Northern Sweden MONICA, incident diagnoses of patients of up to 80 years with myocardial infarction and stroke in the NSHDS are verified by trained staff and detailed clinical information is obtained from patient records. Similar efforts have followed for other disease endpoints, including heart valve disease, venous thromboembolism, atrial fibrillation, and diabetes [5, 15, 17]. Examples of additional cohorts that complement the NSHDS for conducting life course health research are shown in Table 3. The table also provides further examples of registries commonly linked to the NSHDS for research purposes. Notably, additional biological materials, such as archival tumour tissue samples from participants in the NSHDS who developed cancer, can be identified through linkage with pathology records and obtained from the general healthcare biobank (Biobanken Norr). This approach facilitates the analysis of paired pre-diagnostic, diagnostic, and post-diagnostic samples from the same patient in the NSHDS. In addition, for many participants of the NSHDS, genealogical data dating as far back as the eighteenth century are accessible.

**Table 3.** Additional examples of clinical cohorts and registers that are often linked to the NSHDS

Cohort/Register	Description	Reference
The Swedish CArdioPulmonary BioImage Study (SCAPIS)	Population-based cohort studying cardiovascular and chronic obstructive pulmonary diseases.	Bergström G, Berglund G, Blomberg A, *et al.* The Swedish CArdioPulmonary BioImage Study: objectives and design. *J Intern Med.* 2015;278:645–59.
Visualization of asymptomatic atherosclerotic disease for cardiovascular prevention (VIPVIZA)	Randomized trial on visualizing subclinical atherosclerosis for cardiovascular prevention.	Näslund U, Ng N, Lundgren A, *et al.* Visualization of asymptomatic atherosclerotic disease for optimum cardiovascular prevention (VIPVIZA): a pragmatic, open-label, randomised controlled trial. *Lancet*. 2019; 393:133–42.
Uppsala‒Umeå Comprehensive Cancer Consortium (U‒CAN)	Prospective longitudinal biomaterial and clinical data collection of adult cancer patients.	Glimelius B, Melin B, Enblad G, *et al.* U-CAN: a prospective longitudinal collection of biomaterials and clinical information from adult cancer patients in Sweden. *Acta Oncol.* 2018; 57:187–94.
Healthy Ageing Initiative (HAI)	Health examination, risk measurement, and intervention for 70-year-olds in Umeå.	Nordström A, Hadrévi J, Olsson T, Franks PW, Nordström P. Higher prevalence of type 2 diabetes in men than in women is associated with differences in visceral fat mass. *J Clin Endocrinol Metab.* 2016;101:3740–46.
Betula study	Prospective longitudinal study on ageing, memory, and dementia for adults in Umeå.	Nilsson L-G, Adolfsson R, Bäckman L, de Frias CM, Molander B, Nyberg L. Betula: a prospective cohort study on memory, health and aging. *Aging Neuropsychol Cogn.* 2004;11:134-148.
NorthPop database and biobank	Population-based birth cohort of pregnant women, partners, and children in Västerbotten.	https://www.northpop.se/en/home-2/ (accessed 18 September 2024)
The Northern Sweden Maternity cohort	Longitudinal cohort of >125 000 serum samples from >90 000 pregnant women in Västerbotten.	Lukanova A, Toniolo P, Zeleniuch-Jacquotte A, *et al.* Insulin-like growth factor I in pregnancy and maternal risk of breast cancer. *Cancer Epidemiol Biomarkers Prev.* 2006;15:2489–93.
National Patient Register	Contains specialist care data like diagnoses, procedures, dates for admissions and discharges.	Ludvigsson JF, Andersson E, Ekbom A, *et al.* External review and validation of the Swedish national inpatient register. *BMC Public Health.* 2011;11:450.
The Swedish Cause of Death Register	Contains data with regard to the underlying and contributing causes of death.	Brooke HL, Talbäck M, Hörnblad J, *et al.* The Swedish cause of death register. *Eur J Epidemiol.* 2017;32(9):765–73.
National Prescribed Drug Register	Contains data like age, sex, prescribed and dispensed medication, and prescriber's profession.	Wettermark B, Hammar N, Fored CM, *et al.* The new Swedish Prescribed Drug Register—opportunities for pharmacoepidemiological research and experience from the first six months. *Pharmacoepidemiol Drug Saf.* 2007;16:726–35.
The longitudinal integrated database for health insurance and labour market studies (LISA)	Contains sociodemographic and socioeconomic data: civil status, migration, income, employment.	Ludvigsson JF, Svedberg P, Olén O, Bruze G, Neovius M. The longitudinal integrated database for health insurance and labour market studies (LISA) and its use in medical research. *Eur J Epidemiol.* 2019;34:423–37.

A recent initiative, PREDICT (Personalized screening, risk prediction, and understanding disease trajectories for early detection of disease—An integrated cohort approach) aims to promote collaborative and resource-efficient use of the VIP. Currently under development, PREDICT involves 50 274 participants of the VIP who provided a blood sample before 1 January 2011, and either are deceased or gave new consent. Within PREDICT, a subcohort of 7500 individuals, representative of the background population, was established, enabling case-cohort studies of different diseases. In close cooperation with the NSHDS, PREDICT will serve as a research infrastructure, providing a data lake of pooled large-scale data and biochemical analysis results.

## What has it found?

The NSHDS enabled hundreds of publications and >60 PhD theses (https://www.umu.se/en/biobank-research-unit/about-the-unit/publications-and-validations-studies), with only a selection of findings presented here. The impact of the VIP on population health was investigated in multiple studies. A cohort analysis with >1 million person-years observed approximately a 10% reduction in all-cause mortality compared to the Swedish population [18]. Another study observed improved health outcomes in screening detected type 2 diabetes patients within the VIP compared to clinically detected type 2 diabetes patients [19]. In addition, in Västerbotten, blood pressure, fasting glucose levels, and smoking cessation exhibited faster improvement rates than in the neighbouring region [20], whereas the improvement of dietary habits was minimal in comparison [21]. Other findings based on the NSHDS provided early evidence for associations of viruses with subsequent disease risk. For example, associations were identified between Epstein-Barr virus and multiple sclerosis [22], herpes simplex virus and Alzheimer’s disease [23], and human papillomavirus and cervical cancer (crucial for later vaccine development and corresponding screening strategies) [24–26]. More recent studies have supported the significance of the availability of prospective longitudinal samples and/or longitudinal population data to better understand marker-disease associations in, for example, type 2 diabetes [27], cardiovascular disease [28], spondyloarthritis [29], and B cell lymphoma [30]. The NSHDS has also contributed to large international efforts, such as the development of risk prediction models for prevention of cardiovascular disease [31] and early lung cancer detection [32].

## What are the main strengths and weaknesses?

The large size of the NSHDS, comprising >140 000 participants who provided >240 000 high-quality blood samples along with 1.5 million person-years of follow-up over four decades, are the major strengths of the cohort. Together with the high proportion of participants with repeated sampling occasions, and the accessibility of comprehensive health, lifestyle, and clinical data, the NSHDS has a unique position among prospective cohorts. Other strengths include the population-based design of the NSHDS, the complete integration of the VIP into the general healthcare system [10], and the broad consent in the population with high participation [33], all of which help reduce selection bias. Nevertheless, some differences in socio-demographic factors like age and marital status were observed between the NSHDS participants and non-participants in previous studies [10–12]. Such detailed information was not available for the present study. The blood sample quality, particularly in the VIP and the MONICA, is high and has been used for mRNA extraction from buffy coat for gene expression studies, even without the use of an RNA preservative [34]. One limitation of the NSHDS is the lack of cell-cryopreservation, which prevents cell-based analyses. The investigation of rare diseases can be limited due to sample size but is often conducted in collaboration with other cohorts such as EPIC [7]. Additionally, as in other epidemiological studies [35], participation in the VIP and the MONICA has declined in the recent years (Fig. 2 and Supplementary Fig. S1).

## Can I get hold of the data? Where can I find out more?

The use of the NSHDS data and samples is encouraged. Access is covered by Good Biobank Practice, a system for quality control [36]. Studies are usually performed in collaboration with local researchers. Expert groups assess the scientific value of all incoming research proposals. Contact with the Section of Biobank and Registry Support at Umeå University is encouraged at the planning stage (info.brs@umu.se). Application details are provided online (https://www.umu.se/en/biobank).

## Ethics approval

In the VIP, data and blood samples are collected within general healthcare, and informed consent is obtained separately for the research blood sample. In the MSP and the MONICA, informed consent is obtained for data and blood samples. Information given in consent forms changed over time to comply with ethical and legal requirements. All research utilizing the NSHDS must be approved by the Swedish Ethical Review Authority (reference number 2021-03226 for this report) and must comply with the Declarations of Helsinki and Taipei. All data handling must comply with the European General Data Protection Regulation.

## Use of artificial intelligence (AI) tools

Microsoft Copilot was used to improve the readability and to check English grammar of this manuscript.

## Supplementary Material

dyaf004_Supplementary_Data

## Data Availability

See ‘Can I get hold of the data?’ section.
