# Supplementary material for: Cohort Profile: The Northern Sweden Health and Disease Study (NSHDS)
Source: Int J Epidemiol. 2025 Feb 3;54(1):dyaf004. doi: 10.1093/ije/dyaf004 (PMC11790227; doi:10.1093/ije/dyaf004)

**Supplementary Material**

**Supplementary Figure S1.** Participation in the Northern Sweden Monitoring of Trends and Determinants of Cardiovascular Disease (MONICA) Study


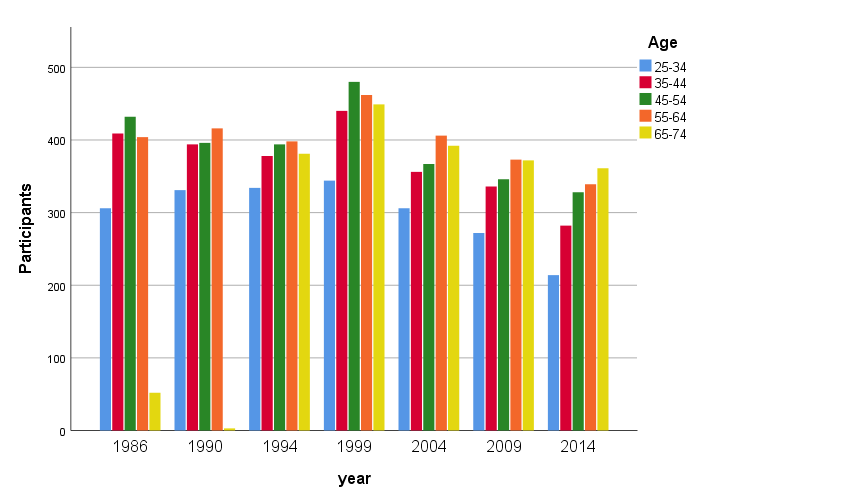

Supplement: dyaf004_Supplementary_Data [file dyaf004_supplementary_data.zip › 1acd1_ije-2024-06-0908-File005.docx]
